# Hydroxymethylfurfural Formation and Sensory Implications in Raisins: Effects of Cultivar, Extra Virgin Olive Oil Pretreatment, and Storage Temperature

**DOI:** 10.3390/plants15101440

**Published:** 2026-05-08

**Authors:** Victoria Diniz Shimizu-Marin, Danilo Henrique Bruno, Yara Paula Nishiyama-Hortense, Carolina Olivati, Ellen Silva Lago-Vanzela

**Affiliations:** Institute of Biosciences, Humanities and Exact Sciences (Ibilce), São Paulo State University (UNESP), Cristóvão Colombo 2265, São José do Rio Preto 15054-000, Brazil; victoria.shimizu@unesp.br (V.D.S.-M.); danilo.bruno@unesp.br (D.H.B.); yara.nishiyama@unesp.br (Y.P.N.-H.); carolinaolivati@gmail.com (C.O.)

**Keywords:** Maillard reaction, non-enzymatic browning, BRS Clara, BRS Vitoria, shelf life, food quality

## Abstract

Hydroxymethylfurfural (HMF) is a well-established marker of heat-induced reactions in sugar-rich foods. However, its accumulation in raisins in response to clean-label pretreatments and its association with consumer sensory perception remain unclear. This study investigated the influence of extra virgin olive oil (EVOO) pretreatment, cultivar (BRS Clara and BRS Vitoria), and storage conditions (4, 25, and 35 °C) on HMF formation in raisins and on their sensory implications. Cultivar influenced physicochemical properties, but neither cultivar nor pretreatment significantly affected HMF levels after drying. During storage, HMF formation followed predominantly zero-order reaction kinetics, with Q10 values (7.33–8.39) confirming strong temperature dependence, and pronounced accumulation at 35 °C. Sensory analysis showed that flavor was the main driver of consumer perception, with burnt sugar notes more frequently cited as a disliked attribute in samples stored at higher temperatures, whereas samples stored at lower temperatures retained attributes closer to time zero. Pearson correlation analysis confirmed a strong positive association between HMF and the burnt sugar descriptor (r = 0.86, *p* < 0.001). These findings demonstrate that EVOO pretreatment can improve drying efficiency without promoting HMF formation, and highlight the value of the combined chemical–sensory approach to assess quality changes in raisins from tropical-adapted grape cultivars.

## 1. Introduction

Food systems are at the center of the interactions between human health, environmental sustainability, and socio-economic resilience, with the One Health concept playing a fundamental role in understanding and integrating these dimensions [1,2]. In this context, fruits are essential components of healthy and sustainable diets. However, their seasonality and high perishability impose limitations on regular accessibility, highlighting the importance of food processing as a strategy to extend shelf life and reduce losses along the food supply chain [3,4]. Dried fruits, including raisins, represent a traditional and commercially relevant approach to enhancing fruit stability, reducing losses, and improving accessibility within sustainable food systems [5,6]. Raisin quality is shaped by a complex interaction among genetic factors, edaphoclimatic conditions, maturity stage, pretreatments, processing, and storage conditions [5,7].

**Figure 1:**
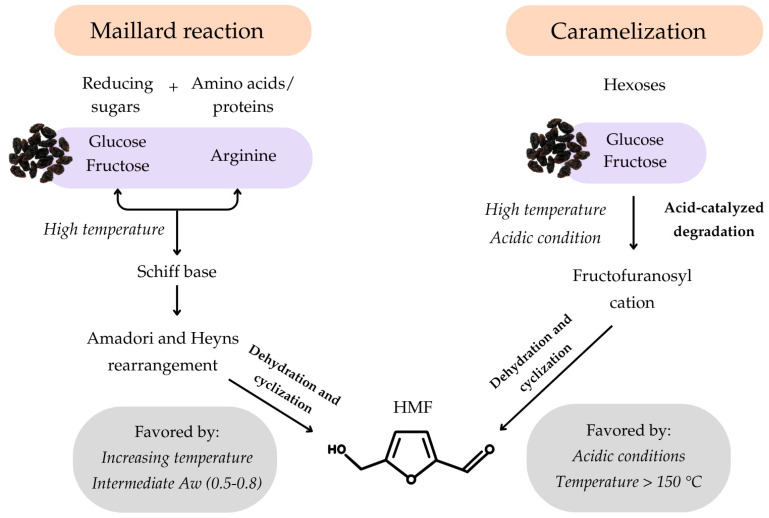
Schematic representation of the chemical pathways of 5-hydroxymethylfurfural (HMF) formation via the Maillard reaction and caramelization, highlighting key precursors, intermediates, and favorable conditions. Based on El Hosry et al. [13], Martins et al. [14], Choudhary et al. [15], Chan et al. [16], and Aktag et al. [17].

During grape dehydration and subsequent raisin storage, non-enzymatic browning reactions may occur as a consequence of heat exposure, progressive water loss, and sugar concentration, leading to significant changes in chemical composition and sensory attributes [7,8,9,10]. A key marker of these reactions is 5-hydroxymethylfurfural (HMF), an intermediate compound that can be formed through two main pathways (Figure 1). In the Maillard reaction pathway, reducing sugars such as glucose and fructose present in grapes react with free amino groups of amino acids—particularly arginine, one of the most abundant and reactive amino acids in grapes [11,12,13]—to form a Schiff base, which rearranges to produce Amadori and Heyns products. These intermediates undergo dehydration and cyclization to yield HMF, a process favored by increasing temperature and time, moderate water activity (0.5–0.8), and the presence of free amino acids [13,14,15]. In the caramelization pathway, under acidic conditions and thermal treatment, hexoses—especially fructose—undergo acid-catalyzed degradation to form a fructofuranosyl cation, which is subsequently converted to HMF by further dehydration and cyclization [14,15,16]. Both pathways are favored by elevated temperatures, low moisture, and acidic conditions, characteristics inherent to raisins, and may occur simultaneously during processing and storage [17].

Previous studies have demonstrated that HMF accumulation is influenced by the intrinsic composition of the fruit, as well as by processing parameters and storage conditions. Gürsul Aktağ and Gökmen [9] reported that fruits with lower pH exhibited higher HMF levels, with raisins (pH 3.79) presenting markedly greater HMF concentrations than dried dates (pH 6.61), underscoring the role of acidity in promoting sugar dehydration reactions. Additionally, surveys of dried fruits have shown that HMF levels are generally higher in dehydrated products than in fruit juices or purées, highlighting the susceptibility of low-moisture, sugar-rich matrices to heat-induced transformations [9]. Although dried fruits possess intrinsic characteristics that favor HMF formation, such as high reducing sugar and hexose concentrations, low moisture, and pH, and the influence of temperature on HMF formation is well established, its occurrence and technological relevance in dried fruits remain less explored than in other sugar-rich products such as honey, fruit juices, syrups, and concentrates [13,14,15]. In addition, most approaches have focused on occurrence, processing conditions, or safety-related aspects rather than on the combined interpretation of technological practices, chemical stability, and sensory perception. In this sense, the novelty of this study is not merely to confirm the temperature dependence of HMF formation, but to determine whether processing strategies adopted to improve drying efficiency may unintentionally affect HMF accumulation and consumer-perceived quality.

This knowledge gap is particularly relevant for recently developed cultivars adapted to tropical conditions, whose technological performance still requires comprehensive characterization. Genotype plays a decisive role in grape chemical composition and, consequently, the reactions induced during processing and storage. In Brazil, breeding programs conducted by the Brazilian Agricultural Research Corporation (Embrapa, Brasília, Brazil) have led to the development of seedless grape cultivars adapted to tropical and subtropical climates, such as BRS Clara (BC) and BRS Vitoria (BV) [18,19]. These cultivars differ in soluble solids content, acidity, and phenolic profile, which may influence drying kinetics and the formation of non-enzymatic browning-derived compounds. However, their suitability for raisin production under an integrated perspective of technological quality, chemical stability, and sensory quality remains insufficiently explored.

Additionally, pretreatment strategies may alter skin permeability and water removal rates, thereby influencing processing time and the formation of thermally induced reaction products. In dried apricots, for example, sulfuring was the most influential factor affecting HMF accumulation, followed by storage time and drying method [20]. Edible oils, particularly extra virgin olive oil (EVOO), have emerged as clean-label alternatives to traditional alkaline treatments used to accelerate drying [21,22,23]. Owing to their high content of antioxidant compounds, these oils may interfere with oxidative and non-enzymatic browning reactions. Nevertheless, the extent to which EVOO pretreatment affects HMF formation and its subsequent evolution during storage remains unclear.

Within this framework, there is a need for an integrated approach that simultaneously considers plant matrix composition, technological variables, chemical stability, and sensory perception. Despite the growing body of research on HMF in processed foods, studies specifically addressing its formation and evolution in raisins during storage, and particularly its association with consumer sensory perception, remain scarce. Furthermore, the potential of clean-label pretreatments such as EVOO to modulate HMF formation in raisins has not been previously investigated. Therefore, the present study aimed to evaluate the formation and evolution of HMF in raisins produced from Brazilian seedless grape cultivars, investigating the influence of EVOO pretreatment, genotype, and storage conditions. To the best of our knowledge, this is the first study to simultaneously quantify HMF accumulation in raisins over time at different storage temperatures and to correlate these chemical findings with consumer sensory perception, providing an integrated assessment of quality changes in dried fruit systems. Furthermore, the evaluation of EVOO as a clean-label pretreatment strategy represents an additional contribution to the development of more sustainable and consumer-oriented raisin production approaches.

## 2. Results and Discussion

### 2.1. Effect of EVOO Pretreatment on Drying Efficiency

**Figure 2:**
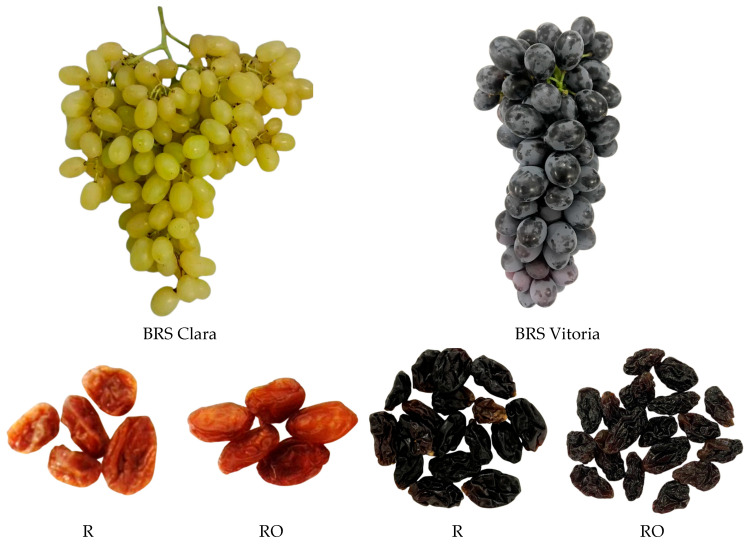
BRS Clara and BRS Vitoria grapes and their corresponding raisins obtained without (R) and with (RO) extra virgin olive oil pretreatment.

The results of the first part of the study showed that the use of EVOO as a pretreatment markedly influenced dehydration efficiency. BC and BV-H1 grapes dried without pretreatment required total drying times of 47 and 70 h, respectively. The grapes and their respective raisins obtained after drying are shown in Figure 2. When the same cultivars were subjected to EVOO pretreatment, drying times were reduced to 27 h for BC and 47 h for BV-H1. Thus, the application of the surfactant agent resulted in approximately 43% and 33% reductions in drying time for BC and BV-H1, respectively.

This expressive reduction indicates that EVOO effectively facilitates dehydration, promoting faster and more efficient water transfer. The effectiveness of this pretreatment can be attributed to the chemical composition of EVOO, which is rich in fatty acids that interact with the lipid components of the grape cuticular wax, a naturally occurring protective layer on the fruit surface. According to Shimizu-Marin et al. [23], scanning electron microscopy analyses demonstrated that the surfactant action of olive oil promotes the formation of micropores on the fruit surface and may induce partial collapse of cells and intermolecular bonds within the plant tissue. As a result, resistance to water diffusion is reduced, facilitating moisture migration from the interior to the surface of the berries and accelerating drying.

Consistent trends have been reported for the same cultivars used in the present study. Olivati et al. [22] evaluated the drying of BC grapes across three harvest seasons and reported reductions in drying time of 34% to 45% when EVOO was applied as a pretreatment. Similarly, Shimizu-Marin et al. [23] reported that the same pretreatment reduced the drying time of BV grapes by approximately 26%. These findings reinforce the reproducibility and effectiveness of EVOO as a natural pretreatment for enhancing dehydration efficiency in these cultivars.

### 2.2. Effect of EVOO Pretreatment and Cultivar on Physicochemical Characteristics and HMF Formation in Raisins

The physicochemical characteristics of the grapes are presented in Table 1. The length values obtained for BC grapes in the present study were close to those reported by Lago-Vanzela et al. [24], who described dimensions of 20 mm in length and 15 mm in width. BC grapes showed significantly greater length (*p* ≤ 0.05) compared to BV-H1, while no significant differences were observed in width or weight between the cultivars. For BV-H1, the measured dimensions (21.8 mm in length and 15.7 mm in width) were within the range reported by Maia et al. [19] and Garcia-Santos et al. [25], who described values ranging from 19 to 22.8 mm for length and 15.32 to 18.15 mm for width.

BC grapes presented significantly lower total acidity (TA), resulting in a correspondingly higher pH value (*p* ≤ 0.05). However, for both cultivars, the pH values were lower than those reported in the literature. Lago-Vanzela et al. [24] and Mascarenhas et al. [26] described pH values of 3.85 and 3.92 for BC, respectively, while Martineli et al. [27] and Garcia-Santos et al. [25] reported values ranging from 3.85 to 4.06 for BV. Regarding TA, the results for both grapes comply with the recommendation proposed by Mascarenhas et al. [26], who suggest that table grapes should not exceed 1.5 g tartaric acid∙100 g^−1^.

The soluble solids (SS) content of BC grapes was significantly higher (*p* ≤ 0.05) than that of BV-H1. These values are consistent with the ranges reported in the literature for BC (18.66–18.9 °Brix) [24,26] and BV (17.33–18.92 °Brix) [25,27]. Although the SS content of BV-H1 was below the range considered ideal for consumption (19.0–23.0 °Brix), as reported by Maia et al. [19], it still falls within the limits permitted by international commercialization standards (14.0–17.5 °Brix). The higher SS content combined with the lower TA of BC resulted in a maturity index (SS/TA ratio) more than 75% higher than that of BV-H1 and also higher than values previously reported in the literature (26.7 and 35.1) [24,26]. Maia et al. [19] emphasize that fruit flavor is largely determined by the balance between sugars and organic acids, and that the SS/TA ratio is more representative of sensory quality than isolated measurements of these parameters. According to these authors, higher maturity index values are desirable in the domestic market, and table grapes should present values above 20, a requirement fulfilled by both cultivars evaluated in this study.

Finally, BV-H1 grapes exhibited higher (*p* ≤ 0.05) moisture content than BC grapes, which may have contributed to the longer drying time observed for grapes processed both with and without EVOO pretreatment. Overall, the physicochemical characteristics of both cultivars were consistent with literature data and within the recommended quality standards for table grapes, confirming that the fruits were in adequate condition for raisin processing.

The mean physicochemical results for the raisins are presented in Table 2, and the analysis of variance results evaluating the effects of cultivar, EVOO pretreatment, and their interaction are shown in Table 3. All raisins obtained complied with national and international quality standards, respecting the maximum moisture limit of 25% established by Brazilian legislation [28]. The values also met the requirements of CODEX STAN 360-2020 [29], which establishes that moisture content in raisins must not exceed 18%.

**Figure 3:**
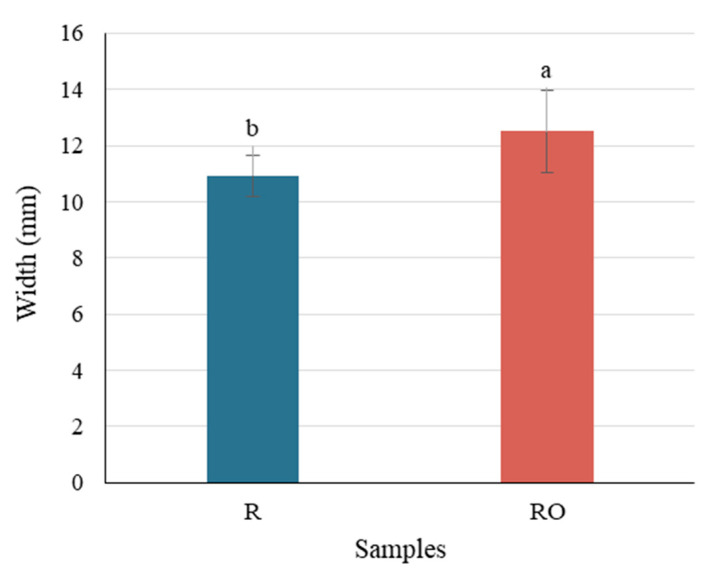
The main effect of pretreatment on raisin width obtained by two-way ANOVA. Values are mean ± standard deviation; different letters indicate significant differences (*p* ≤ 0.05). R = raisins without pretreatment; RO = raisins with EVOO pretreatment.

Among the evaluated parameters, ANOVA indicated that only width was significantly influenced by the pretreatment, with pretreated raisins (RO) presenting greater dimension (Figure 3) than untreated ones (R). This effect was supported by a large effect size (η^2^ = 0.41) and moderate statistical power (1 − β = 0.64). This behavior may indicate that faster drying promoted by the pretreatment may contribute to better preservation of structural integrity, minimizing tissue collapse during dehydration and consequently reducing lateral shrinkage.

**Figure 4:**
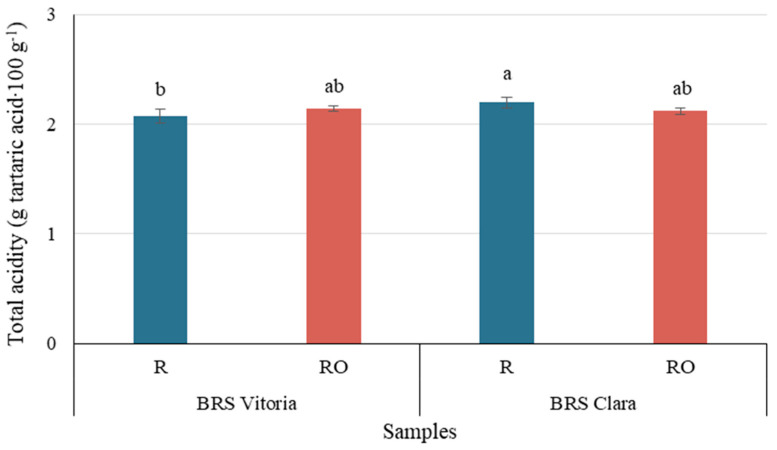
Effect of pretreatment and cultivar interaction on total acidity of raisins obtained by two-way ANOVA. Values are mean ± standard deviation; different letters indicate significant differences (*p* ≤ 0.05). R = raisins without pretreatment; RO = raisins with EVOO pretreatment.

TA was the only parameter significantly affected by the interaction between cultivar and pretreatment (*p* ≤ 0.05), indicating that the response to EVOO depended on grape genotype. This interaction was supported by a large effect size (η^2^ = 0.53) and adequate statistical power (1 − β = 0.84), reinforcing the reliability of this finding. In R samples, BC raisins showed higher TA than BV-H1, whereas no significant differences were observed between R and RO within each cultivar (Figure 4). The RO samples showed intermediate values (Table 2) that did not differ statistically from their respective controls. These findings suggest that EVOO pretreatment did not directly reduce TA, but rather modulated it depending on cultivar-specific characteristics.

Similar behavior was reported by Olivati et al. [22] for BC, in which significant differences were observed in only one of three harvest seasons, indicating that the effect of oil-based pretreatments on acidity is not consistent. Reports for BRS Vitoria also suggest a cultivar-dependent response [23]. As the final moisture content did not differ significantly among treatments, the variations in TA cannot be attributed solely to concentration effects. Instead, differences in acid stability during drying or genotype-specific matrix characteristics may explain the observed interaction. From a technological perspective, these variations may influence sugar–acid balance and flavor perception in raisins.

For the remaining physicochemical parameters (moisture, pH, length, and weight) no significant effects of pretreatment, cultivar, or their interaction were observed (*p* > 0.05). Effect size and statistical power analyses provided additional insight into the interpretation of these non-significant findings. For all parameters, the pretreatment factor yielded negligible to small effect sizes (η^2^ ranging from 0.02 to 0.17) with consistently low statistical power (1 − β ≤ 0.24), suggesting that the absence of significant effects genuinely reflects a lack of meaningful influence of EVOO pretreatment on these characteristics under the evaluated conditions. In contrast, cultivar presented large effect sizes for moisture (η^2^ = 0.38) and pH (η^2^ = 0.30), despite not reaching statistical significance, with moderate statistical power (1 − β of 0.59 and 0.45, respectively), suggesting that cultivar-related differences in these parameters may exist but were not fully detected, probably given the number of replicates available.

The HMF content obtained for all samples (Table 2) was close to that reported by Gürsul Aktağ et al. [9], who found HMF concentrations of 189.5 mg·kg^−1^ in Sultana-type raisins pretreated with hot water and dried at 70 °C. No significant effects of cultivar, pretreatment, or their interaction were observed (*p* > 0.05) (Table 3). Effect size and statistical power analyses further supported these findings, as all factors yielded negligible to small effect sizes (η^2^ = 0.003, 0.02, and 0.11 for pretreatment, cultivar, and their interaction, respectively) with low statistical power (1 − β = 0.058, 0.109, and 0.350, respectively), indicating that the absence of significant effects is consistent with a genuine lack of meaningful influence of these factors on HMF formation under the evaluated conditions. These results suggest that the reduction in drying time promoted by EVOO pretreatment was not sufficient to significantly influence HMF formation during raisin processing. From a mechanistic standpoint, the EVOO pretreatment acts as a surfactant, dispersing the naturally present epicuticular wax layer on the grape surface, thereby promoting the formation of micropores that facilitate moisture migration and reduce drying time [23]. However, this structural modification primarily affects water-transfer resistance at the surface, without directly altering the thermal conditions experienced by the berry interior during drying. It is likely that drying temperature exerts a greater influence on HMF formation than the time of heat exposure, and therefore the accelerated drying promoted by EVOO pretreatment may not be sufficient to substantially modify HMF accumulation at a constant drying temperature of 60 °C. Although the effect of drying temperature on HMF formation was not evaluated in the present study, this interpretation is consistent with the findings of previous studies indicating that drying temperature is the dominant driver of HMF accumulation in dried fruit products. Gürsul Aktağ et al. [20], for instance, reported higher HMF concentrations in oven-dried apricots at 65 °C than in sun-dried samples, despite the longer drying time required for sun drying. Similarly, Wojdyło et al. [30] observed that increasing the drying temperature from 50 to 70 °C during convective drying led to greater HMF accumulation, even though the drying time was reduced from 9 to 7 h. This effect was attributed to the higher temperature reached by the material during drying, which showed an exponential relationship with HMF formation. In addition, a threshold temperature of approximately 60 °C was identified, above which HMF formation increased rapidly. These findings reinforce that temperature is a key driver of HMF formation, outweighing the effect of reducing drying time.

Although the evaluated cultivars differed in physicochemical characteristics, particularly TA, these variations did not result in significant differences in HMF formation. Since acidic conditions may favor sugar dehydration reactions involved in HMF generation, the absence of cultivar effects suggests that the compositional differences between BC and BV-H1 were not sufficient to substantially influence the formation of this compound under the processing conditions applied. Overall, these findings indicate that, within the evaluated conditions, drying temperature likely plays a more decisive role in HMF formation than moderate differences in grape composition or the application of EVOO pretreatment.

### 2.3. Effects of Storage Time, Temperature, and Pretreatment on HMF Formation in BRS Vitoria Raisins

In the second part of the study, BV-H2 raisins presented HMF contents of 0.24 mg⋅g^−1^ for R samples and 0.21 mg⋅g^−1^ for RO samples immediately after processing (day 0). These values were similar to those previously obtained for BV-H1 raisins (Table 2, Section 2.2), reinforcing the consistency of the experimental procedure. To the best of our knowledge, no previous study has simultaneously monitored HMF accumulation and its association with consumer sensory perception in raisins stored under controlled temperature conditions, making the findings presented in this section a novel contribution to the understanding of quality changes in dried fruit systems.

**Figure 5:**
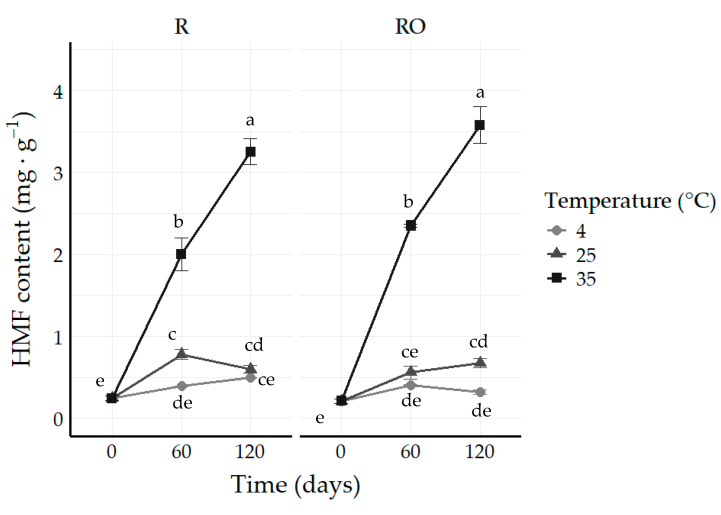
Effect of pretreatment, storage temperature, and time interaction on 5-hydroxymethylfurfural (HMF) content of raisins obtained by three-way ANOVA. Values are mean ± standard deviation; different letters indicate significant differences by Tukey’s test (*p* ≤ 0.05). R = raisins without pretreatment; RO = raisins with EVOO pretreatment.

During storage, ANOVA revealed that HMF formation was significantly affected by the interaction between pretreatment, storage time, and temperature (*p* ≤ 0.05). The effects of this interaction are presented in Figure 5. No significant differences were observed among samples at day 0, corroborating the results obtained in the first part of the study. These findings further support the conclusion that EVOO pretreatment, although effective in reducing drying time, does not influence the HMF content formed during raisin processing.

The pronounced accumulation of HMF during storage at elevated temperatures is consistent with the known chemistry of its formation. Under the evaluated storage conditions, both the Maillard reaction and caramelization pathways may, in principle, contribute to HMF generation. The Maillard pathway, which involves the reaction of reducing sugars with amino groups, is favored by elevated temperatures, intermediate water activity, and the presence of free amino acids—conditions characteristic of stored raisins [9,13]. The caramelization pathway, in turn, involves the acid-catalyzed degradation of fructose under acidic conditions and thermal treatment to form a fructofuranosyl cation, which is subsequently converted to HMF by further dehydration and cyclization [15]. However, caramelization-derived HMF formation is generally reported to be favored at temperatures above 150 °C [14,15], well above the storage conditions evaluated in the present study. Therefore, although the relative contribution of each pathway cannot be determined from the present data alone, the combination of elevated temperatures, intermediate water activity, high reducing sugar content, and the presence of free amino acids strongly suggests that the Maillard reaction pathway is the predominant route of HMF formation under the conditions evaluated, consistent with findings reported for other dried fruit matrices [9].

At storage temperatures of 4 and 25 °C, HMF contents showed only modest changes. Shapla et al. [31] report that HMF can form even at relatively low temperatures under acidic or low-pH conditions, which may help explain the increase observed in raisins stored at 25 °C. At this temperature, a significant increase in HMF content was observed after 60 days compared to day 0 (*p* ≤ 0.05). However, HMF levels did not differ significantly from those observed at 4 °C at the same storage time. These results indicate a slow progression of non-enzymatic browning reactions under refrigerated and ambient conditions during the evaluated period.

In contrast, storage at 35 °C resulted in a pronounced and progressive increase in HMF content over time. At this temperature, both R and RO raisins exhibited significantly higher HMF levels after 60 days compared to samples stored at 4 and 25 °C (*p* ≤ 0.05). After 120 days, HMF contents increased markedly, reaching 3.26 mg⋅g^−1^ for R samples and 3.58 mg⋅g^−1^ for RO samples, representing an increase of more than tenfold relative to the initial values. These results demonstrate that elevated storage temperature markedly accelerates HMF formation in raisins, regardless of the pretreatment. It should be noted, however, that given the limited specificity of the thiobarbituric acid colorimetric method, other carbonyl-containing compounds formed during prolonged storage at elevated temperatures may also contribute to the absorbance signal at 443 nm, potentially leading to a partial overestimation of HMF content under these conditions [14,32].

The effect of storage time on the increase in HMF content in fruit products has been reported by other authors, such as Udomkun et al. [33] for dried papaya and Gürsul Aktağ [20] for dried apricots. Similar trends have also been observed in other dried fruits. Aktag et al. [9], for instance, quantified HMF in dates, raisins, and blueberries during 6 months of storage and reported the highest concentration in raisins at the end of the period (4151.1 mg·kg^−1^). This behavior is consistent with the findings of Shapla et al. [31], who reported that higher storage temperatures combined with prolonged storage times strongly influence HMF accumulation in food products.

The kinetic parameters of HMF formation during storage are presented in Table 4. At 35 °C, the zero-order model provided the best fit for both R and RO raisins (R^2^ = 0.9909 and 0.9763, respectively), suggesting that HMF accumulation proceeded at an approximately constant rate under this condition. At 25 °C, the zero-order model provided a good fit for RO raisins, but not for R raisins, for which both models yielded low R^2^ values. At 4 °C, however, a good model fit was observed only for R raisins. These results likely reflect the small magnitude of HMF variation observed under these conditions, as previously discussed, which limits the robustness of the kinetic fitting.

The activation energy (Ea) estimated by the Arrhenius equation under the zero-order model was higher for RO (Ea = 72.78 kJ·mol^−1^) compared to R (Ea = 49.06 kJ·mol^−1^). It should be noted, however, that the Arrhenius model fit for R was relatively weak (R^2^ = 0.667), in contrast to the satisfactory fit obtained for RO raisins (R^2^ = 0.900); therefore, the kinetic parameters derived for R raisins should be interpreted with caution. Nonetheless, the higher Ea observed for RO raisins indicates that HMF formation in EVOO-pretreated samples requires more energy to overcome the energy barrier and drive the reaction, but exhibits greater temperature sensitivity across the evaluated range. This higher Ea may be partly attributed to the antioxidant compounds naturally present in EVOO, which may scavenge reactive intermediates involved in the Maillard reaction, thereby increasing the energy threshold required to initiate HMF formation [13]. Additionally, structural modifications of the berry surface induced by the pretreatment, including the formation of micropores [23], may influence oxygen permeability and favor oxidative reactions involved in HMF accumulation at elevated temperatures. However, these remain speculative hypotheses, and further analyses would be required to confirm the underlying mechanisms. Q10 values of 7.33 and 8.39 for RO and R raisins, respectively, confirmed the strong temperature dependence of HMF formation in the 25–35 °C range for both treatments, with R raisins showing slightly higher sensitivity in this specific interval.

The predominance of zero-order kinetics observed in the present study is consistent with findings reported in the literature for other food matrices. Rattanathanalerk et al. [34] observed that HMF increased linearly with time in thermally treated pineapple juice across a wide temperature range (55–95 °C), supporting the zero-order behavior identified here. Similarly, Karadeniz et al. [35] reported zero-order kinetics for HMF formation in date fruit filling during storage. The Q10 values obtained in the present study (7.33 and 8.39 for RO and R raisins, respectively) are higher than those reported by Karadeniz et al. [35] for date fruit filling (5.11 and 7.90 for the temperature ranges of 25–35 °C and 35–45 °C, respectively), suggesting that HMF formation in raisins is particularly sensitive to temperature increases in the 25–35 °C range, which has direct implications for the definition of appropriate storage conditions for this product.

### 2.4. Evolution of Consumer Sensory Perception of BRS Vitoria Raisins During Storage

**Figure 6:**
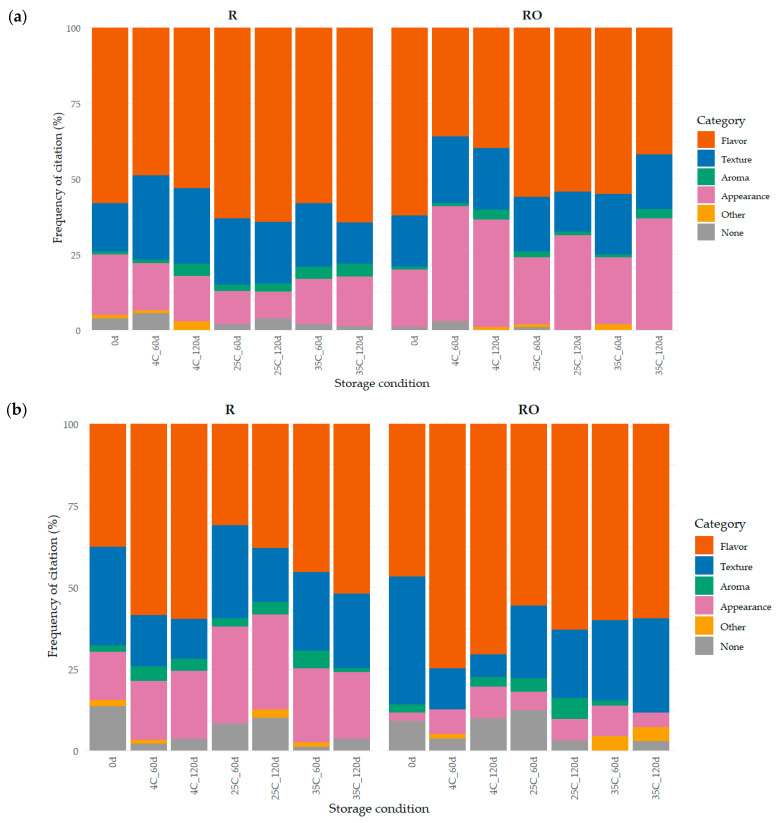
Frequency of consumer citations (%) of main sensory categories mentioned as: (**a**) most liked and (**b**) least liked for raisins without (R) and with (RO) extra virgin olive oil pretreatment during storage at 4, 25, and 35 °C (60 and 120 days). The 0 d sample corresponds to the baseline prior to storage (time zero).

The frequency of citation (%) obtained from the open-ended questions, considering the attributes mentioned as “most liked” and “least liked”, is presented in Figure 6a and Figure 6b, respectively. The results obtained were categorized into four main sensory groups: flavor, texture, aroma, and appearance. In addition to the main sensory categories, the category “Other” comprised descriptors that could not be clearly assigned to a specific sensory dimension, including affective terms (e.g., “tasty”) and low-frequency mentions. The category “None” corresponded to responses in which participants explicitly indicated that no attribute was perceived as liked or disliked.

Regarding the terms cited as “least liked”, flavor was again the most frequently mentioned category across all samples, reinforcing its central role not only as a driver of liking but also of rejection. For R, flavor-related negative terms ranged from 31.0% (25 °C, 60 days) to 59.8% (4 °C, 120 days), while for RO samples, frequencies varied from 46.8% (time 0) to 74.7% (4 °C, 60 days). These results suggest that flavor changes over storage were critical in determining consumer dissatisfaction, particularly under prolonged refrigerated conditions.

The Correspondence Analysis (CA) of the open-ended responses (“What did you like most?” and “What did you dislike most?”) revealed a consistent influence of storage temperature and time on the sensory perceptions reported by consumers. The responses were categorized by sensory attribute, enabling visualization of associations between samples and the terms most frequently mentioned by participants. For the “What did you like most?” question, Dimensions 1 and 2 explained 31.6% and 30.1% of the total inertia, respectively. For the “What did you dislike most?” responses, the first two dimensions explained 40.8% and 24.0% of the total inertia. Together, the maps demonstrate that storage conditions not only modified the dominant positive attributes perceived by consumers but also shaped the nature of the defects reported during storage.

**Figure 7:**
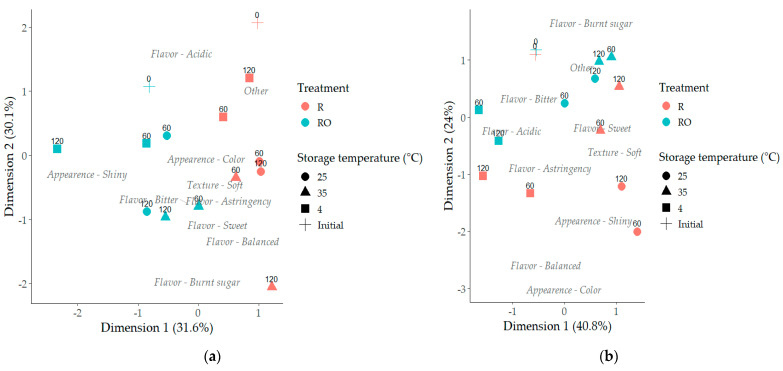
Correspondence analysis (CA) of consumer: (**a**) “most liked” and (**b**) “least liked” responses for raisins without (R) and with (RO) extra virgin olive oil pretreatment subjected to different storage conditions. Numbers represent storage time (0, 60, and 120 days).

The correspondence analysis map of responses to “What did you like most?” (Figure 7a) revealed clear associations between storage conditions and the positive attributes consumers perceived. The descriptor flavor–acidic was positioned close to the initial samples (Time 0), indicating that this characteristic was more noticeable immediately after processing. Among the stored samples, those kept at 4 °C, particularly R, remained relatively close to this attribute, suggesting a better preservation of the original flavor profile during storage at lower temperatures.

In contrast, samples stored at higher temperatures and for longer periods shifted toward descriptors associated with more developed flavor notes. The attribute flavor–burnt sugar, for example, was located near the samples stored at 35 °C for 120 days, especially R. The development of the burnt sugar sensory attribute observed in these samples stored at higher temperatures may be related to non-enzymatic browning reactions occurring during storage. Although caramelization of sugars can contribute to caramel-like flavor notes, similar sensory attributes have also been associated with products of the Maillard reaction, which generate volatile compounds responsible for caramelized, roasted, and burnt sensory perceptions in thermally processed foods [36]. It is important to highlight that compounds associated with sweet, burnt, pungent, and caramel-like aromas are frequently attributed to specific classes of Maillard reaction products, such as furans, furanones, and pyranones, to which HMF belongs [13,37]. Other descriptors, such as sweet, balanced, astringent, and bitter, were positioned in the lower region of the map, reflecting changes in flavor perception over storage.

Although the separation was not absolute, a tendency for samples to distribute according to pretreatment was also observed, with RO positioned more toward the left side of the map and R toward the right. This pattern suggests that the pretreatment may have contributed to subtle differences in the sensory profile perceived by consumers, possibly related to changes in the surface characteristics, particularly in relation to the attribute appearance–shiny.

Similar results were observed in the responses to “What did you dislike most?” (Figure 7b). The CA showed that the attributes perceived as negative by consumers varied according to storage conditions and sample characteristics. Flavor-related descriptors such as acidic and astringency were positioned closer to samples stored at 4 °C, suggesting that these sensory perceptions were more frequently mentioned as undesirable under refrigerated storage conditions. This observation is consistent with the pattern identified in the CA of the “liked most” responses, where flavor–acidic was also associated with the initial samples and with raisins stored at lower temperature, indicating that refrigerated storage tended to preserve characteristics closer to those perceived immediately after processing. This highlights the variability in consumer perception and the dual role that certain flavor attributes may play depending on individual preferences.

In addition, R tended to be positioned closer to the descriptor appearance–shiny in the “disliked most” map. In this context, the mention of this attribute reflects the lack or insufficient intensity of surface shine perceived by consumers rather than the presence of this characteristic. This interpretation is supported by the results observed in the correspondence analysis of the “liked most” responses, where appearance–shiny was associated with samples that were positively perceived by consumers. Therefore, the presence of this attribute among the disliked responses suggests that reduced surface brightness negatively influenced consumer perception. Considering that the EVOO pretreatment forms a thin oil layer on the grape surface before drying, the greater shine perceived in these samples may be attributed to the presence of EVOO, which likely contributed to the more glossy appearance of the raisins.

Finally, the descriptor flavor–burnt sugar was positioned closer to samples stored under more severe conditions, particularly those stored at higher temperatures (35 °C) and for longer periods (120 days). A similar association was observed in the CA of the “liked most” responses, where this attribute was also linked to samples stored at elevated temperatures. The recurrence of this descriptor in both analyses suggests that storage conditions promoting more intense chemical changes influenced consumer perception of caramelized or burnt flavor notes. Such sensory characteristics are commonly associated with advanced stages of non-enzymatic browning reactions, including both caramelization and the Maillard reaction, that occur during processing and storage of dried fruit products. These reactions are also related to the formation of compounds such as HMF, whose accumulation was observed under similar storage conditions in the present study (Section 2.3).

**Figure 8:**
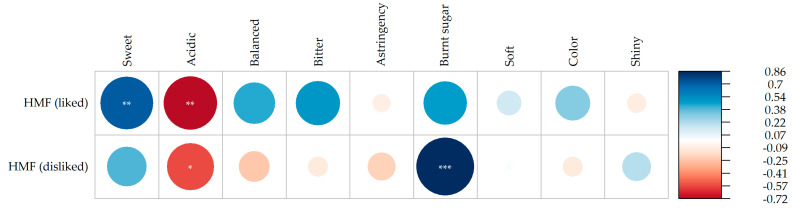
Pearson correlation between HMF content and the frequency of sensory descriptors reported by consumers as most liked and most disliked for BRS Vitoria raisins. Significance levels: * *p* ≤ 0.05; ** *p* ≤ 0.01; *** *p* ≤ 0.001. HMF: 5-hydroxymethylfurfural.

To further explore the relationship between HMF formation and consumer sensory perception, Pearson correlation analysis was performed between HMF content and the frequency of sensory descriptor citations for both “liked most” and “disliked most” responses (Figure 8).

For the “liked most” data, HMF content showed a significant positive correlation with the descriptor sweet (r = 0.71, *p* < 0.01) and a significant negative correlation with acidic (r = −0.72, *p* < 0.01), indicating that as HMF accumulated during storage, consumers tended to appreciate the sweetness of the samples, while acidity, more frequently cited as a positive attribute in initial samples and those stored at lower temperatures, became less appreciated over time. For the “disliked most” data, the most relevant association was the strong positive correlation between HMF and the descriptor burnt sugar (r = 0.86, *p* < 0.001), suggesting that the accumulation of HMF under more severe storage conditions may be directly associated with the development of sensory characteristics perceived as undesirable by consumers. These findings provide a quantitative link between chemical and sensory data, reinforcing HMF as an indicator of quality changes in raisins during storage.

It should be acknowledged that the use of open-ended questions, while effective in capturing spontaneous consumer perceptions in their own words without prior attribute conditioning, presents inherent limitations. The categorization of verbatim responses into sensory attributes requires manual interpretation by the researcher, which can be time-consuming and may introduce subjectivity, particularly when responses are ambiguous or when synonymous terms need to be grouped [38]. Furthermore, this method does not allow quantification of sensory intensity, limiting the depth of analytical comparisons compared to more structured methodologies [38]. Future studies may benefit from complementary approaches, such as check-all-that-apply (CATA) questions, to provide a more comprehensive and less interpreter-dependent characterization of sensory attributes.

## 3. Materials and Methods

### 3.1. Plant Material and Experimental Design

Lots of the cultivars BC and BV (named BV-H1) were obtained from the Tropical Viticulture Experimental Station of EMBRAPA, located in Marialva, Paraná State, Brazil. Part of each lot was processed into raisins using two different conditions: (i) without EVOO pretreatment (control, R) and (ii) with EVOO pretreatment (RO), as described in Section 2.2. Fresh grapes and the corresponding raisins were subjected to physicochemical characterization and to the determination of HMF.

A second independent lot of BV (named BV-H2) grapes was obtained from vineyards located in Marinópolis, São Paulo State, Brazil. These grapes were also processed into raisins (R and RO) and used exclusively for HMF evolution and sensory analysis during storage.

**Figure 9:**
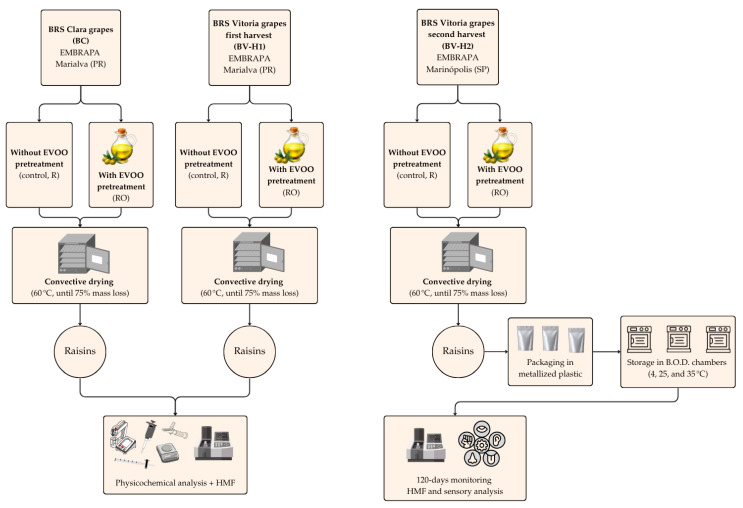
Flowchart of the experimental design and overall workflow of the study. EVOO = Extra virgin olive oil; HMF = hydroxymethylfurfural.

A flowchart was developed to visually represent the experimental design and facilitate understanding of the study’s overall structure and workflow (Figure 9).

### 3.2. Raisin Processing and EVOO Pretreatment

Grapes were initially sanitized by immersion in an aqueous solution containing 0.5% (*v*/*v*) active chlorine for 20 min, followed by rinsing with potable water and natural drainage. Subsequently, half of the berries were subjected to pretreatment with EVOO (acidity ≤ 0.50% and peroxide value ≤ 20.00 milliequivalents⋅kg^−1^), applied through manual homogenization at a concentration of 0.2% (m/m), according to the procedure described by Olivati et al. [21]. The oil was weighed directly into a container, and the grapes were then added on top to minimize mass transfer losses. Manual mixing was performed until all berries were uniformly coated, as visually confirmed by the transition from the characteristic whitish appearance of the epicuticular wax layer to a uniform glossy surface. This visual criterion was applied consistently across all replicates to ensure application uniformity.

The dehydration process was performed in a convective dryer (TE-394/2, Tecnal, São Paulo, Brazil) with hot air circulation at 60 °C and an air velocity of 1 m⋅s^−1^. Drying progress was monitored by periodic weighing of the trays, and dehydration was continued until the berries reached at least a 75% reduction in their initial mass.

### 3.3. Physicochemical Analyses

Previously defined portions of the samples (BC and BV-H1 fresh grapes and raisins R and RO) were used for physicochemical analyses according to the official methods of the Association of Official Analytical Chemists [39]. Moisture content was determined by the gravimetric method. SS were quantified only in fresh grapes by refractometry (RTA-100, Quimib, São Paulo, Brazil), and results were expressed as °Brix at 25 °C. The pH was measured directly using a calibrated digital pH meter (Tec-5, Tecnal, Piracicaba, Brazil) that had been previously standardized with buffer solutions at pH 4.0 and 7.0. TA was determined by potentiometric titration and expressed as g of tartaric acid⋅100 g^−1^.

The SS/TA ratio was calculated as the quotient between SS and TA. In addition, berries and raisins were evaluated for their dimensions (length × width, cm) using a digital caliper (King Tools, Shanghai, China), and for mass (g) using an analytical balance (AY220, Shimadzu, Barueri, Brazil). All physicochemical analyses were performed in triplicate.

### 3.4. Determination of 5-Hydroxymethylfurfural

The content of HMF was determined according to the method proposed by Keeney and Bassette [40] and adapted by Rattanathanalerk, Chiewchan, and Srichumpoung [34]. Briefly, 1 g of the raisin sample was homogenized with 15 mL of 90% acetone solution using an Ultra-Turrax homogenizer (T18 Digital, IKA, Staufen, Germany) for 1 min, followed by centrifugation at 12,000× *g* for 2 min. The supernatant was collected as the extract.

An aliquot of 2 mL of the extract was mixed with 2 mL of 12% (m/m) trichloroacetic acid and 2 mL of 0.025 M thiobarbituric acid. The reaction mixture was incubated in a water bath at 40 °C for 50 min, and then cooled to room temperature, and the absorbance was measured at 443 nm using a UV–Vis spectrophotometer (Agilent Technologies Cary 60 UV-Vis, Santa Clara, CA, USA).

Quantification was performed using an external calibration curve prepared with analytical-grade HMF standard (Merck, Germany), ranging from 5.0 × 10^−6^ to 9.0 × 10^−5^ mol⋅L^−1^, with a coefficient of determination of R^2^ = 0.9984. The limit of detection (LOD) and limit of quantification (LOQ) were 4.07 × 10^−6^ mol⋅L^−1^ and 1.23 × 10^−5^ mol⋅L^−1^, respectively. Method repeatability, assessed by triplicate analysis of samples, yielded coefficients of variation (CV) ranging from 0 to 14%, with a mean CV of 6.7%, which is considered acceptable for complex food matrices such as raisins. Results were expressed as mg of HMF⋅g of raisin^−1^.

It should be noted that the reaction with thiobarbituric acid serves as a derivatization step that enhances the selectivity of the method by shifting the measurement to 443 nm in the visible region, where common matrix interferents absorb considerably less than at 284 nm, the wavelength used for direct UV detection of HMF [14]. Nevertheless, other carbonyl-containing compounds may still react with thiobarbituric acid under the described conditions, and results should be interpreted with this limitation in mind.

### 3.5. Monitoring of HMF and Sensory Evaluation During Storage

For the study of HMF evolution and sensory analysis during storage, BV-H2 raisins (R and RO) were packed in stand-up pouch packages (approximately 125 × 80 mm), made of double-layer metalized plastic (PET + PE), characterized by low oxygen permeability (< 60 cm^3^⋅m^−2^⋅day^−1^) and low water vapor transmission (<5 g⋅m^−2^⋅day^−1^). Three replicate packages were prepared for each treatment and storage temperature. Packaged raisins were stored in B.O.D. incubators (CE-300/350-FAU, Cienlab, Campinas, Brazil) at 4, 25, and 35 °C, with approximately 50% relative humidity, for 120 days. HMF quantification and sensory analysis were performed for both R and RO raisins at storage times of 0, 60, and 120 days.

The kinetics of HMF formation during storage were modeled by fitting the experimental data to zero-order and first-order reaction models, described by the standard Equations (1) and (2), respectively:

C = C_0_ + k·t (1)

C = C_0_ · e ^(k·t)^ (2)

where C is the HMF content at time t (days), C_0_ is the initial HMF content (day 0), and k is the reaction rate constant (mg HMF⋅g^−1^⋅day^−1^ for zero-order reaction and day^−1^ for first-order reaction). The best-fit model was selected based on the coefficient of determination (R^2^). The temperature dependence of the rate constants was described by the Arrhenius equation (Equation (3)):

k = A · e^(−Ea/RT)^ (3)

where Ea is the activation energy (kJ·mol^−1^), A is the pre-exponential factor, R is the universal gas constant (8.314 J·mol^−1^·K^−1^), and T is the absolute temperature (K). The Ea was estimated from the slope of the linear regression of ln(k) versus 1/T.

The sensory study was approved by the Research Ethics Committee of the Institute of Biosciences, Humanities and Exact Sciences (IBILCE), São Paulo State University (UNESP), São José do Rio Preto campus (CAAE: 58114322.6.0000.5466; approval number: 5.450.197). Written informed consent was obtained from all participants prior to the study. A total of 90 raisin consumers participated in the evaluation. The median age was 23 years (range: 18–49 years). Regarding gender identification, 53% self-identified as women, 42% as men, and 4% as other.

Raisins, R and RO, evaluated under different storage conditions, were served monadically using a balanced complete block design with repeated measures over time. The same 90 consumers evaluated all samples at 0, 60, and 120 days of storage. Samples were coded with random three-digit numbers and presented in a balanced order during each evaluation session to minimize order and carryover effects [41]. Consumers were asked to answer two open-ended questions: (i) “What did you like most about this sample?” and (ii) “What did you like least about this sample?” Water was provided for palate cleansing between samples.

The open-ended responses were transcribed and grouped into sensory categories and subcategories based on semantic similarity. The main categories included flavor, texture, aroma, and appearance, each comprising specific subcategories (e.g., sweet, acidic, bitter, astringent, balanced, soft, color, and shine).

Descriptors that could not be clearly assigned to a specific sensory category, as well as affective terms (e.g., “tasty”, “delicious”), were grouped into the “Other” subcategory within their respective question (“most liked” or “least liked”). Additionally, descriptors mentioned by a low number of consumers were also incorporated into the “Other” category to avoid excessive fragmentation of the data. Participants who explicitly wrote “nothing” were classified under the category “None”. Blank responses were excluded from the frequency calculation. Therefore, citation frequencies were calculated considering only the number of valid responses for each sample and evaluation time.

### 3.6. Statistical Analysis

All statistical analyses were performed using R software [42]. Data were evaluated by analysis of variance (ANOVA) to assess the effects of treatment, cultivar, and their interaction on HMF content and physicochemical parameters. For storage experiments, a factorial ANOVA was applied to evaluate the effects of treatment, storage temperature, storage time, and their interactions. When significant differences were observed (*p* ≤ 0.05), Tukey’s test was used for multiple comparisons. To support the interpretation of non-significant findings, partial eta-squared (η^2^) was calculated as a measure of effect size using the effectsize package [43], and statistical power (1 − β) was estimated using the pwr package [44]. Effect sizes were interpreted according to the benchmarks proposed by Cohen [45]: η^2^ < 0.01 = negligible, 0.01–0.06 = small, 0.06–0.14 = medium, and >0.14 = large. Kinetic modeling of HMF formation during storage was performed by applying linear regression to fit zero-order and first-order reaction models, and the temperature dependence of rate constants was described by the Arrhenius equation.

For sensory analysis results, citation frequencies were calculated as the percentage of consumers who mentioned each category for each treatment and storage condition. Stacked bar charts were constructed to illustrate the distribution of attributes identified as “most liked” and “least liked”. CA was applied to the contingency table of subcategories to explore associations between samples and sensory descriptors. Data manipulation and organization were performed using the dplyr package [46], while graphical representations were generated using ca package [47] and ggplot2 [48].

## 4. Conclusions

This study demonstrates that processing and storage conditions are key determinants of HMF formation and raisin quality. EVOO pretreatment effectively reduced drying time without significantly affecting HMF formation during processing. Likewise, no significant influence of cultivar or pretreatment on HMF levels was observed immediately after drying, suggesting that the reduction in drying time achieved by EVOO pretreatment does not affect HMF formation under the drying conditions used in this study (60 °C). During storage, HMF accumulation was strongly influenced by temperature and time, with significantly higher values observed at 35 °C. Kinetic modeling confirmed that HMF formation followed predominantly zero-order reaction kinetics, with activation energies and Q10 values indicating strong temperature dependence. These results confirm that elevated storage temperatures accelerate non-enzymatic browning reactions in raisins, directly impacting product quality. Sensory analysis corroborated these findings, as attributes associated with caramelized or burnt flavors became more pronounced under more severe storage conditions, whereas samples stored at lower temperatures retained sensory characteristics closer to those observed immediately after processing.

In this context, HMF can be considered an important indicator of chemical changes associated with non-enzymatic browning and quality loss in dried fruits. The present study, to the best of our knowledge, represents the first integrated assessment of HMF accumulation kinetics and consumer sensory perception in raisins stored under different temperature conditions, thereby filling a gap in the literature on quality monitoring of dried fruit products. The novelty of this study does not lie in demonstrating that temperature promotes HMF formation, but rather in showing that a clean-label EVOO pretreatment can improve drying efficiency without increasing HMF formation, while the combined chemical–sensory approach reveals how storage-driven HMF accumulation relates to consumer-perceived quality changes in raisins from tropical-adapted Brazilian grape cultivars. Additionally, the evaluation of EVOO as a clean-label pretreatment strategy contributes to the body of knowledge on natural and sustainable alternatives for raisin processing, even though its application did not significantly affect HMF formation during drying. The results highlight the importance of optimizing processing conditions and adopting appropriate storage strategies to maintain chemical stability and sensory quality. Furthermore, the use of natural pretreatments, such as EVOO, emerges as a promising approach aligned with the concept of One Health, contributing to the development of safer foods produced through more sustainable and integrated practices that link food quality, human health, and production systems.

## Figures and Tables

**Table 1:** Physicochemical characterization (mean ± standard deviation) of BRS Clara and BRS Vitoria fresh grapes.

Parameter	BRS Clara	BRS Vitoria
Length (mm)	25.2 ± 0.06 *	21.8 ± 0.16
Width (mm)	15.0 ± 0.23	15.7 ± 0.03
Weight (g)	3.04 ± 0.24	3.87 ± 0.58
TA (g tartaric acid∙100 g^−1^)	0.32 ± 0.02	0.45 ± 0.01 *
pH	3.10 ± 0.04 *	3.00 ± 0.02
Moisture (%)	74.39 ± 0.07	82.83 ± 0.10 *
Soluble solids (°Brix)	19.03 ± 0.06 *	15.23 ± 0.06
Maturation index	59.5	33.8

* Indicates a statistically higher mean according to Student’s *t*-test (*p* ≤ 0.05). TA: Total acidity.

**Table 2:** Physicochemical characterization and HMF content (mean ± standard deviation) of BRS Clara and BRS Vitoria raisins without pretreatment (R) and with EVOO pretreatment (RO).

Parameter ^1^	BRS Clara		BRS Vitoria	
	R	RO	R	RO
Length (mm)	21.7 ± 0.10	21.3 ± 0.14	19.8 ± 0.28	23.5 ± 0.09
Width (mm)	10.7 ± 0.08	11.8 ± 0.13	11.2 ± 0.08	13.2 ± 0.16
Weight (g)	1.10 ± 0.08	1.13 ± 0.18	1.05 ± 0.16	1.28 ± 0.25
TA (g tartaric acid∙100 g^−1^)	2.20 ± 0.05	2.12 ± 0.03	2.07 ± 0.06	2.14 ± 0.02
pH	3.38 ± 0.05	3.40 ± 0.04	3.46 ± 0.09	3.46 ± 0.06
Moisture (%)	12.15 ± 0.39	13.23 ± 0.38	14.53 ± 0.28	13.96 ± 0.56
HMF (mg HMF∙g^−1^)	0.187 ± 0.054	0.250 ± 0.118	0.265 ± 0.094	0.220 ± 0.050

^1^ TA: Total acidity; HMF: 5-hydroxymethylfurfural.

**Table 3:** Values of *p*, F-ratio, size effect (η^2^), and statistical power (1 − β) from the two-way ANOVA for the physicochemical characteristics and HMF content of raisins produced with and without extra virgin olive oil pretreatment from the grape cultivars BRS Clara and BRS Vitoria.

Factors		HMF Content ^1^	Moisture	Total Acidity	pH	Length	Width	Weight
Pretreatment	*p*-value	0.798	0.727	0.845	0.733	0.134	**0.045**	0.240
	F-ratio	0.067	0.131	0.041	0.125	2.778	5.641	1.613
	Size effect	0.003	0.020	0.005	0.020	0.260	0.410	0.170
	Statistical power	0.058	0.065	0.055	0.064	0.375	0.644	0.240
Cultivar	*p*-value	0.487	0.057	0.076	0.101	0.872	0.206	0.638
	F-ratio	0.501	4.953	4.163	3.445	0.028	1.891	0.239
	Size effect	0.020	0.380	0.340	0.300	0.004	0.190	0.030
	Statistical power	0.109	0.589	0.518	0.447	0.053	0.273	0.077
Pretreatment × Cultivar	*p*-value	0.131	0.271	**0.017**	0.733	0.081	0.549	0.373
	F-ratio	2.481	1.400	9.032	0.125	4.000	0.391	0.892
	Size effect	0.110	0.150	0.530	0.020	0.330	0.050	0.100
	Statistical power	0.349	0.215	0.836	0.064	0.503	0.095	0.154

^1^ HMF: 5-hydroxymethylfurfural. Bold values indicate statistically significant differences (*p* ≤ 0.05).

**Table 4:** Kinetic parameters of HMF ^1^ formation in BRS Vitoria raisins produced without (R) and with (RO) extra virgin olive oil pretreatment during storage at different temperatures, fitted to zero-order and first-order reaction models.

Sample	Temperature (°C)	Zero-Order				First-Order			
		R^2^	k (mg⋅g^−1^⋅day^−1^)	Ea (kJ⋅mol^−1^)	Q10	R^2^	k (day^−1^)	Ea (kJ⋅mol^−1^)	Q10
R	4	0.9868	0.0021	49.06	8.39	0.9585	0.0059	25.87	2.44
R	25	0.4286	0.0030			0.5481	0.0076		
R	35	0.9909	0.0252			0.8846	0.0217		
RO	4	0.3015	0.0009	72.78	7.33	0.3877	0.0035	41.81	2.85
RO	25	0.9168	0.0038			0.8627	0.0097		
RO	35	0.9763	0.0281			0.8585	0.0236		

^1^ HMF: 5-hydroxymethylfurfural; R^2^: coefficient of determination of the kinetic model fit; k: reaction rate constant; Ea: activation energy. R^2^ values for the Arrhenius model fit were: Zero-order: R = 0.667, RO = 0.900 and First-order: R = 0.711, RO = 0.964; Q10: temperature coefficient calculated for the 25–35 °C interval.

## Data Availability

The original contributions presented in this study are included in the article. Further inquiries can be directed to the corresponding author.

## Abbreviations

The following abbreviations are used in this manuscript:

HMF	5-hydroxymethylfurfural
EVOO	Extra virgin olive oil
BC	BRS Clara grapes
BV	BRS Vitoria grapes
BV-H1	BRS Vitoria grapes from the first harvest
BV-H2	BRS Vitoria grapes from the second harvest
R	Raisins without extra virgin olive oil pretreatment
RO	Raisins with extra virgin olive oil pretreatment
R^2^	Coefficient of determination
k	Reaction rate constant
Ea	Activation energy
Q10	Temperature coefficient
CA	Correspondence Analysis

## References

[1] Bremner J., Vial F., Fooks A., Higman W., Avant J., Stentiford G. (2023). Operationalizing “One Health” for food systems. One Earth.

[2] Lopez Cervantes P., Fernandez Xicotencatl R.I., McCoy Cador C., Kinney I.S. (2024). Circular economy and food safety: A focus on One Health. Appl. Food Res..

[3] Michel M., Eldridge A.L., Hartmann C., Klassen P., Ingram J., Meijer G.W. (2024). Benefits and challenges of food processing in the context of food systems, value chains and sustainable development goals. Trends Food Sci. Technol..

[4] Noor Mohammed A., Chauhan O.P., Semwal A.D. (2024). Emerging technologies for fruits and vegetables dehydration. Food Humanit..

[5] Jeszka-Skowron M., Zgola-Grzeskowiak A., Stanisz E., Waskiewicz A. (2017). Potential health benefits and quality of dried fruits: Goji fruits, cranberries and raisins. Food Chem..

[6] Samtiya M., Aluko R.E., Dhewa T., Moreno-Rojas J.M. (2021). Potential health benefits of plant food-derived bioactive components: An overview. Foods.

[7] Khiari R., Zemni H., Mihoubi D. (2019). Raisin processing: Physicochemical, nutritional and microbiological quality characteristics as affected by drying process. Food Rev. Int..

[8] Angulo O., Fidelibus M.W., Heymann H. (2007). Grape cultivar and drying method affect sensory characteristics and consumer preference of raisins. J. Sci. Food Agric..

[9] Gürsul Aktağ I., Gökmen V. (2021). Investigations on the formation of α-dicarbonyl compounds and 5-hydroxymethylfurfural in fruit products during storage: New insights into the role of Maillard reaction. Food Chem..

[10] Wu Y., Liu Y., Jia Y., Feng C.-H., Zhang H., Ren F., Zhao G. (2024). Effects of thermal processing on natural antioxidants in fruits and vegetables. Food Res. Int..

[11] Gutiérrez-Escobar R., Aliaño-González M.J., Cantos-Villar E. (2024). Variety and year: Two key factors on amino acids and biogenic amines content in grapes. Food Res. Int..

[12] Lima M.M.M., Choy Y.Y., Runnebaum R.C. (2024). Comprehensive amino acids profiling: Grape varieties grown in California and in Tuscany, Italy. J. Food Sci..

[13] El Hosry L., Elias V., Chamoun V., Halawi M., Cayot P., Nehme A., Bou-Maroun E. (2025). Maillard Reaction: Mechanism, Influencing Parameters, Advantages, Disadvantages, and Food Industrial Applications: A Review. Foods.

[14] Martins F.C.O.L., Alcantara G.M.R.N., Silva A.F.S., Melchert W.R., Rocha F.R.P. (2022). The role of 5-hydroxymethylfurfural in food and recent advances in analytical methods. Food Chem..

[15] Choudhary A., Kumar V., Kumar S., Majid I., Aggarwal P., Suri S. (2021). 5-Hydroxymethylfurfural (HMF) formation, occurrence and potential health concerns: Recent developments. Toxin Rev..

[16] Chan A.C., Mokhtar S.U., Hong P.K. (2025). A systematic review on the determination and analytical methods for furanic compounds in caramel models. J. Food Sci. Technol..

[17] Gürsul Aktağ I., Gökmen V. (2020). A survey of the occurrence of α-dicarbonyl compounds and 5-hydroxymethylfurfural in dried fruits, fruit juices, puree and concentrates. J. Food Compos. Anal..

[18] Embrapa Uva e Vinho (2014). Opções de Cultivares de Uva de Mesa Desenvolvidas Pela Embrapa.

[19] Maia J.D.G., Ritschel P.S., Camargo U.A., Souza R.T., Fajardo T.V.M., Naves R.L., Girardi C.L. (2012). BRS Vitória’: Nova Cultivar de Uva de Mesa Sem Sementes Com Sabor Especial e Tolerante ao Míldio.

[20] Gürsul Aktağ I., Doğan Cömert E., Gökmen V. (2026). Effects of sulfuring, drying and storage on the formation of α-dicarbonyl compounds and 5-hydroxymethylfurfural in dried apricots. J. Food Compos. Anal..

[21] Olivati C., Nishiyama Y.P.O., de Souza R.T., Janzantti N.S., Mauro M.A., Gomes E., Hermosín-Gutiérrez I., da Silva R., Lago-Vanzela E.S. (2019). Effect of the pretreatment and the drying process on the phenolic composition of raisins produced with a seedless Brazilian grape cultivar. Food Res. Int..

[22] Olivati C., Nishiyama Y.P.O., Silva R., Gómez-Alonso S., Lago-Vanzela E.S. (2022). BRS Clara raisins production: Effect of the pretreatment and the drying process on the phenolic composition. J. Food Compos. Anal..

[23] Shimizu-Marin V.D., Nishiyama-Hortense Y.P., Olivati C., Gonçales A.C., Garcia-Santos M.S.L., Janzantti N.S., Taboga S.R., Lago-Vanzela E.S. (2024). Natural pretreatment in raisins production: Effects on quality during storage. J. Stored Prod. Res..

[24] Lago-Vanzela E.S., da Silva R., Gomes E., García-Romero E., Hermosín-Gutiérrez I. (2011). Phenolic composition of the Brazilian seedless table grape varieties BRS Clara and BRS Morena. J. Agric. Food Chem..

[25] Garcia-Santos M.S.L., Shimizu-Marin V.D., Nishiyama-Hortense Y.P., Olivati C., de Souza R.T., da Silva F.B., Janzantti N.S., Lago-Vanzela E.S. (2025). BRS Vitória grapes across four production cycles: Morphological, mineral, and phenolic changes. Plants.

[26] Mascarenhas R.J., de Melo Silva S., Coêlho de Lima M.A., Mendonça R.M.N., Holschuh H.J. (2012). Characterization of maturity and quality of Brazilian apirenic grapes in the São Francisco river Valley. Food Sci. Technol..

[27] Martineli M., Mendes F.T., Santos J.R.P., Maranhão C.M.A., Castricini A. (2018). Sensory and quality assessment of processed raisins from three cultivars produced in the semiarid region of Brazil. Braz. J. Food Technol..

[28] Brazil Agência Nacional de Vigilância Sanitária (ANVISA). Resolução RDC nº 726, de 1° de Julho de 2022. https://anvisalegis.datalegis.net/action/UrlPublicasAction.php?acao=abrirAtoPublico&num_ato=00000726&sgl_tipo=RDC&sgl_orgao=RDC/DC/ANVISA/MS&vlr_ano=2022&seq_ato=002&cod_modulo=134&cod_menu=1696.

[29] (2020). General Standard for Dried Fruits. Codex Alimentarius Commission 2020.

[30] Wojdyło A., Lech K., Nowicka P. (2020). Effects of different drying methods on the retention of bioactive compounds, on-line antioxidant capacity and color of the novel snack from red-fleshed apples. Molecules.

[31] Shapla U.M., Solayman M., Alam N., Khalil M.I., Gan S.H. (2018). 5-hydroxymethylfurfural (HMF) levels in honey and other food products: Effects on bees and human health. Chem. Cent. J..

[32] Imperiale S., Morozova K., Ferrentino G. (2022). Fast Detection of 5-Hydroxymethylfurfural in Dulce de Leche by SPE-LC–MS. Food Anal. Methods.

[33] Udomkun P., Nagle M., Argyropoulos D., Mahayothee B., Latif S., Müller J. (2016). Compositional and functional dynamics of dried papaya as affected by storage time and packaging material. Food Chem..

[34] Rattanathanalerk M., Chiewchan N., Srichumpoung W. (2005). Effect of thermal processing on the quality loss of pineapple juice. J. Food Eng..

[35] Karadeniz F., Atalay D., Erge H.S., Kaya S., Işık B., Aslanali O. (2024). Kinetics of 5-hydroxymethylfurfural (5-HMF) formation and colour change in date fruit fillings stored at different temperatures. J. Food Compos. Anal..

[36] Starowicz M., Zieliński H. (2019). How Maillard reaction influences sensorial properties (color, flavor and texture) of food products?. Food Rev. Int..

[37] Mirza Alizadeh A., Mohammadi M., Hashempour-Baltork F., Hosseini H., Shahidi F. (2025). Process-induced toxicants in food: An overview on structures, formation pathways, sensory properties, safety and health implications. Food Prod. Process. Nutr..

[38] Piqueras-Fiszman B. (2015). Open-ended questions in sensory testing practice. Rapid Sensory Profiling Techniques and Related Methods.

[39] AOAC (2012). Official Methods of Analysis of the Association of Official Analytical Chemists.

[40] Keeney M., Bassette R. (1958). Detection of intermediate compounds in the early stages of browning reaction in milk products. J. Dairy Sci..

[41] Macfie H.J.H., Bratchell N., Greenhoff K., Vallis L.V. (1989). Designs to balance the effect of order of presentation and first-order carry-over effects in hall tests. J. Sens. Stud..

[42] R Core Team (2023). R: A Language and Environment for Statistical Computing.

[43] Ben-Shachar M., Lüdecke D., Makowski D. (2020). effectsize: Estimation of Effect Size Indices and Standardized Parameters. J. Open Source Softw..

[44] Champely S. (2020). pwr: Basic Functions for Power Analysis. https://CRAN.R-project.org/package=pwr.

[45] Cohen J. (1988). Statistical Power Analysis for the Behavioral Sciences.

[46] Wickham H., François R., Henry L., Müller K., Vaughan D. (2023). dplyr: A Grammar of Data Manipulation. https://CRAN.R-project.org/package=dplyr.

[47] Nenadic O., Greenacre M. (2007). Correspondence Analysis in R, with two- and three-dimensional graphics: The ca package. J. Stat. Softw..

[48] Wickham H. (2016). ggplot2: Elegant Graphics for Data Analysis.

